# Supplementary material for: Chromothripsis during telomere crisis is independent of NHEJ, and consistent with a replicative origin
Source: Genome Res. 2019 May;29(5):737–49. doi: 10.1101/gr.240705.118 (PMC6499312; doi:10.1101/gr.240705.118)
Supplement: Supplemental Material [file supp_gr.240705.118_Supplemental_file_1.zip › contigs/annotated_contigs/DB93/contig.2.DB93_length_764_mean_cov_14.0536649215.docx]

**DB93_length_764_mean_cov_14.0536649215**

```
TGCTTTTCTGCCTCTCATGTTCAAGCTACCCAGCTACCCATTACACATCTGACACATTTCTATTGAGAAAAATCCCTCATAGATCCAAT
 >chr6:40372853-40373261 + E=1e-233
AACAATGATAGATGTCATCGGAATACCTGCCATTAGCAAGAGAAGCTGAAGAGGCAGCAGGGTACAGTGGATATGCACACAGGCTCTGG

AGTCAGACTGTAGGGTTCCAATCCTGGTTTTGCACATACCAGCTGTAATACCTTATCTTGGGCAAGTTTCTTAATCTCTCTGTGTTTGC

TTCCTAGAAATATCCCCTTTCTGTAAAATGGGGCGTTCATAATCTCTGCCTTATAGGGTTGTTATGAGGATTCAAGGAGTCATGATTCA

TAAAGTACCTAGGTCAGTGCCCGGCACAGAGTAAGCCCCGCATGAGGG|TCTG|AGCTGAGGGTTAATCAGAACCAGGGTAGCCACACT
                                                 >chr6:40310452-40310812 + E=1e-201
TCCCTTTCAGATAATTCCAGGGCGGCTCTGAGTCTCTAAAACCTGTGAGAAATGCCACAGCTGGGAGACTTTCCTTTCCTCCTTGATTC

AAGGAAGTCCAAGAGCCAGGGATGGTGCCCCTCACACTGCCAGGAAGGCCTGACACTACCCCTCCCCATGGCGCAAGCTGAGGCCAATC

CGCTCCTTATTTAGAAAGAGCTGGGACTTAAATGTCAGTGGATGATACGGGAGCAGCATGACAATTACCAGCTTAAACTGAAATAAAGG

AAAGAAGGATTAAAAAACACCCAGCACCCATCAAGCCAGGCCAGAAGAGGAGAG
```
